# Analysis of Uncertainty and Repeatability of a Low-Cost 3D Laser Scanner

**DOI:** 10.3390/s120709046

**Published:** 2012-07-02

**Authors:** María-Eugenia Polo, Ángel M. Felicísimo

**Affiliations:** University Centre of Mérida, University of Extremadura, 06800 Mérida, Spain; E-Mail: amfeli@unex.es

**Keywords:** laser scanning, low-cost scanner, uncertainty, repeatability, NextEngine

## Abstract

Portable 3D laser scanners are a valuable tool for compiling elaborate digital collections of archaeological objects and analysing the shapes and dimensions of pieces. Although low-cost desktop 3D laser scanners have powerful capacities, it is important to know their limitations. This paper performs an analysis of the uncertainty and repeatability of the NextEngine™ portable low-cost 3D laser scanner by scanning an object 20 times in two different resolution modes—Macro and Wide. Some dimensions of the object were measured using a digital calliper, and these results were used as the “true” or control data. In comparing the true and the scanned data, we verified that the mean uncertainty in the Macro Mode is approximately half that of the Wide Mode, at ±0.81 mm and ±1.66 mm, respectively. These experimental results are significantly higher than the accuracy specifications provided by the manufacturer. An analysis of repeatability shows that the successive replicates do not match in the same position. The results are better in Macro Mode than in Wide Mode; it is observed that the repeatability factor is slightly larger than the corresponding mode accuracy, with ±0.84 *vs.* ±0.81 mm in Macro Mode and ±1.82 *vs.* ±1.66 mm in Wide Mode. We suggest several improvements, such as adding an external reference scale or providing a calibrated object to allow for a self-calibration operation of the scanner.

## 1. Introduction

Several techniques and devices for acquiring 3D information have been developed in recent years. Depending on the purpose and budget, one can choose between several devices for obtaining 3D data [1]. Terrestrial laser scanners (TLS) allow for obtaining a large amount of 3D positional information in a fast and efficient way. They are used in numerous applications, such as forestry [2], landslides [3], cultural heritage [4,5], deformation analysis [6] and geomorphology [7]. Because of their continuous increasing use, it is crucial to know both the error source and the uncertainty of any TLS measurement range. Several studies have analysed the behaviour of these instruments [8–10].

As terrestrial laser scanners have become more available, their applications have become more widespread, creating a demand for affordable, efficient and user-friendly devices. While some 3D studies require more sophisticated devices, most can be performed with low-cost and small-sized desktop 3D laser scanners. These low-cost 3D laser scanners are becoming useful in a wide range of applications, such as 3D documentation for archaeological studies [11,12], art restoration [13], forensic sciences [14], palaeontology [15] and food process modelling [16]. Therefore, it is very important for users to know the accuracy and uncertainty of these devices [17] and the factors that can influence the quality of 3D scanned data [18].

The objective of this paper is to perform an analysis of the uncertainty and repeatability of the NextEngine™ portable low-cost 3D laser scanner [19], using it to scan the same object several times in two standard resolution modes. This analysis will be conducted in two stages. The first step relies on assessing both the variation of the position and the dimensions of the scanned data in several repetitive scans. The second stage deals with the comparison of the repetitive scanned data in different resolution modes. Finally, several suggestions will be made to improve the features of this type of scanner.

## 2. Materials and Study Case

This study was conducted with a NextEngine™ HD Desktop 3D scanner, a USD $3000 device with external dimensions of 277 × 223 × 91 mm and a weight of 3.2 kg. Additional materials provided by the manufacturer include a turntable and a thin support for unstable objects. The NextEngine™ is a triangulation-based laser scanner designed for scanning small- and medium-sized objects. The scanner utilises twin arrays of four solid-state lasers and twin 3.0 Megapixel CMOS image sensors for texture recording in colour mode.

This desktop 3D scanner works in two modes: Macro and Wide. Each mode presents a different baseline length between laser and sensor and, therefore, a different field of view and accuracy. According to the manufacturer specifications, the accuracy in Macro Mode is ±0.005 inch (±0.13 mm), providing a maximum point density of 400 points per inch. The accuracy in Wide Mode is ±0.015 inch (±0.38 mm) with a point density of 150 points per inch. The field sizes at the standard distances for the Macro and Wide Modes are 130 × 97 mm at 166 mm distance and 345 × 258 mm at 435 mm distance, respectively [20].

The scanner is managed using ScanStudio™ HD PRO, a software program that acquires data and processes the point clouds using various functions, such as merging scans, meshing, simplifying, filling gaps, *etc.*

The scanned object used in the study is a wooden birdhouse with external dimensions of 58 × 140 × 65 mm. We selected this object because wood has a suitable reflective property (moderate noise, absence of glare) and can be easily measured using a calliper (see below).

## 3. Methods

### 3.1. Scanning the Object

Selected dimensions of the wooden birdhouse were measured several times by two operators using a digital calliper (with a specified accuracy of 0.01 mm), and the results were used as the “true” or control data. The measurements were taken between the centres of the sides. Because the sides were not perfectly parallel, the calliper and calibrated gauges were used to guarantee the accurate locations in the centre of each side.

**Figure 1.**
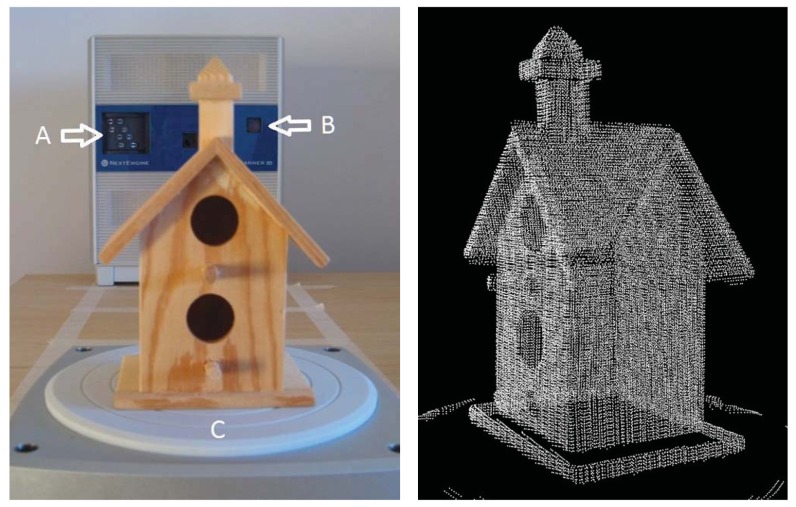
**Left:** A wooden birdhouse was scanned 20 times both in Macro and Wide Mode (40 scans in total) using a motorised turntable (A: laser; B: camera; C: turntable). **Right:** A view of the 3D point cloud generated by the scanner.

The object was scanned 20 times in both Macro and Wide Mode (40 scans in total) (Figure 1). The configuration parameters (full 360° rotation, 7 divisions, 500 points per square inch, neutral target), and the environmental conditions remained constant. No filters and post-processing transformations were applied to study the behaviour of the raw data in terms of repeatability. The raw data were exported as plain text (X, Y, Z coordinates) without texture information. The reference system of the scanner defines the Z axis as depth (perpendicular to scanner front), the Y axis as height, and the X axis as width. All units are in mm.

### 3.2. Measuring the Uncertainty

**Figure 2.**
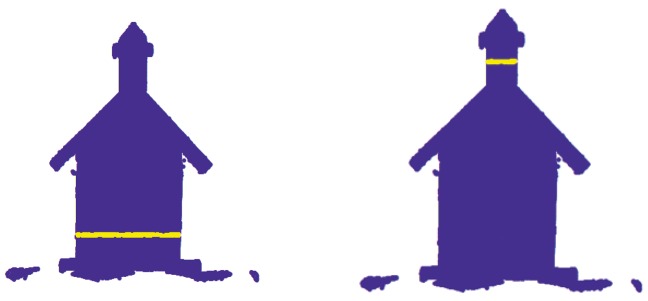
Cross section of the scanned birdhouse obtained from the Geographic Information System. The yellow lines show the position of the two extracted sections for each scan: house body section (B section) on the left and chimney section (C section) on the right.

Two sections for each replicate were extracted in both Macro and Wide Mode (80 sections in total). This process was performed with a Geographic Information System by extracting points from coordinate ranges. This resulted in two rectangular sections in the XZ plane all in the identical zones used for calliper measurement. These sections are called house body section (B section), and chimney section (C section), respectively (Figure 2).

**Figure 3.**
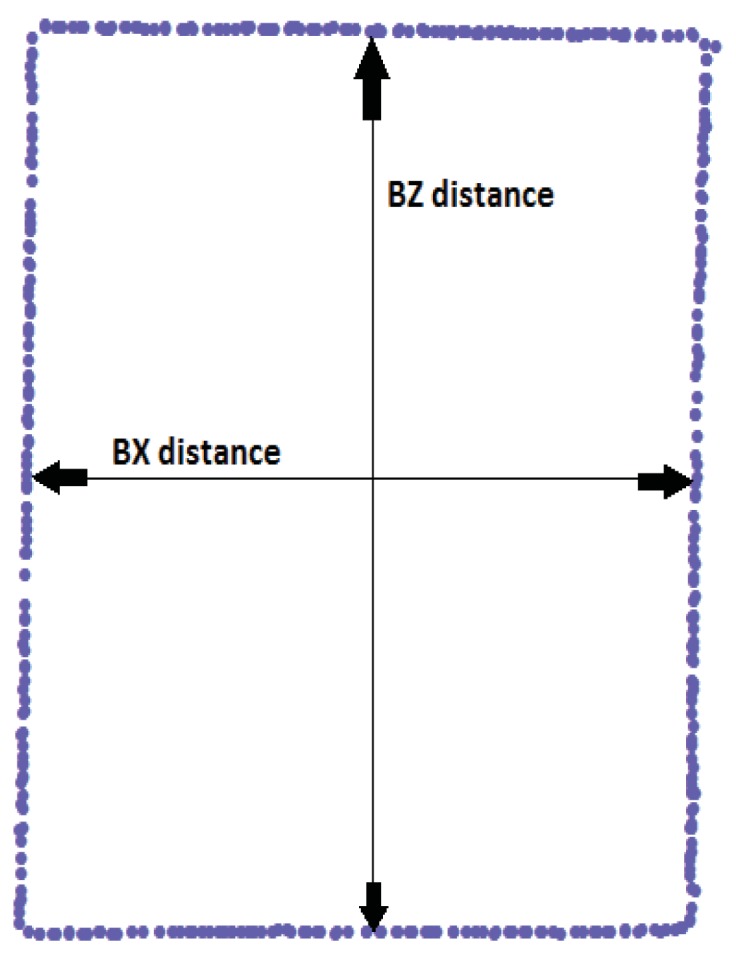
An example of a point section extracted from the point clouds for the B section. Arrows indicate the distances to be measured.

The next step was to measure the distance between the centres of the sides in each point section (Figure 3). Four distances were measured: two in the B section and two in the C section. The four distances are identified by the codes BX, BZ (B section along the X and Z axis) and CX, CZ (C section along the X and Z axis).

When these measurements are performed manually, determining the exact points of measurement can be haphazard, making replicate measurements difficult. To avoid uncertainty and to standardise the measurements, we used the following procedure: (1) the coordinates of the points of each section were imported onto a spread sheet, (2) linear regressions and endpoint coordinates were calculated to define the rectilinear segments that best represent each side, (3) the coordinates of the mean point for each side were calculated from the regression equations and endpoints, and (4) the distances between the opposite mean points were estimated.

This procedure was designed to guarantee that the points obtained from regressions match the points obtained from calliper measurements. At the end of this stage, we have the measurements of distance between each pair of sides in all the replicates. The last step was to compare both sets of measurements and to estimate the uncertainty of the scanner data. One hundred sixty distances were measured in total (4 distances between opposite lines × 20 replicates × 2 modes).

### 3.3. Measuring the Repeatability

**Figure 4.**
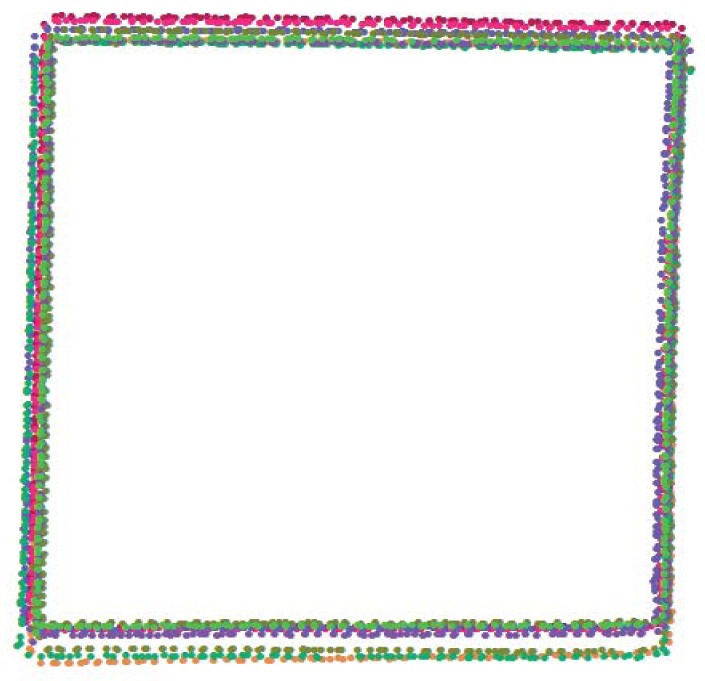
Graphic of ten B sections; the replicates do not match.

The previous test compared the calliper and scanner measurements for estimating the absolute accuracy of the object dimensions. However, throughout the process, it was observed that the replicates did not exactly match in the same position (Figure 4). To analyse this potential problem, a complementary analysis was performed; the intersections of several lines were estimated from the regression equations.

This analysis of repeatability (ability to generate the same coordinates for the same feature in different replicates) can be performed by comparing the coordinates of a recognisable feature or element. We selected the four corners (see Figure 4) to analyse the matching of coordinates. Because visually defining the exact location of the corners from the points proved inaccurate, we decided to define the location of these corners from the regression calculated intersection of the rectilinear segments, as described in the previous section. Thus, 80 corners (four in each replicate) were calculated, both in Macro and Wide Modes, from the B section. The absolute coordinates have been standardised in relation to the local (corner) centroid to allow for comparison in both Macro and Wide Modes (see Section 4.2).

## 4. Results

### 4.1. Measuring the Uncertainty

The digital calliper measurements (mean and 95% confidence interval) are as follows: BX = 58.68 ± 0.07 mm, BZ = 51.11 ± 0.09 mm, CX = 13.25 ± 0.05 mm and CZ = 12.84 ± 0.02 mm (n = 20). Table 1 shows the mean value and the standard deviation obtained from the differences between the true measurement and the scanned data.

A systematic error appears in three of the eight cases from Table 1 involving the Z distances in both Macro and Wide Modes, with the exception of BZ distance in Wide Mode. These results suggest that the measurements along the Z axis are less accurate than those along the X axis and tend to overestimate the actual distances.

The mean uncertainty in the Macro Mode is approximately half that of the Wide Mode: ±0.81 mm and ±1.66 mm, respectively (95% CI: ±1.96 standard deviation). The experimental results are significantly higher than the accuracy data provided in the manufacturer specifications (±0.13 mm in Macro Mode and ±0.38 mm in Wide Mode).

### 4.2. Measuring the Repeatability

**Figure 5.**
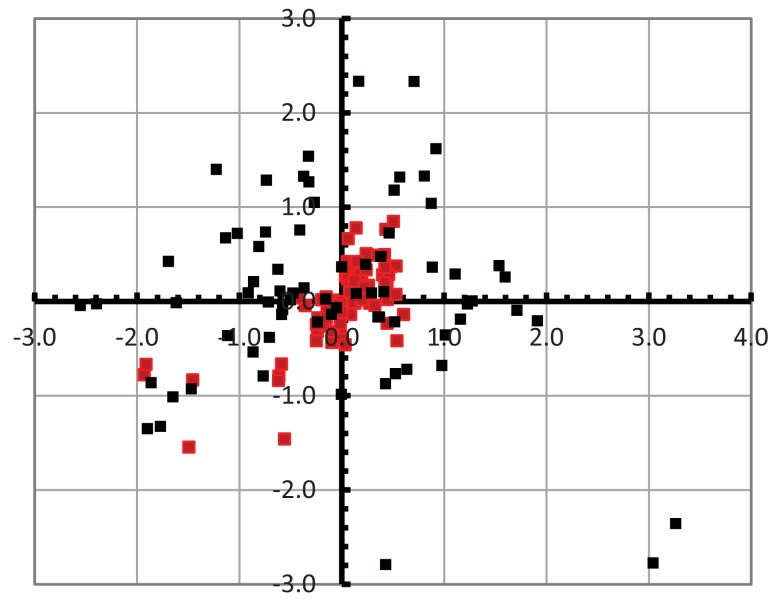
The dispersion of the corners of the B section (in millimiter). The coordinates are relative to the centroid (0, 0). The Wide Mode corners are in black, and the Macro Mode corners are in red.

The level of repeatability is based on the ability of the scanner to place the object at the same location relative to the coordinates of the scanner. We calculated the coordinates of all the corners of the B section and analysed the results using VecStatGraphs2D, an R module developed in our research group [21]. By standardising the absolute coordinates in relation to the local (corner) centroid (0, 0) to allow comparison in both modes, 2D error vectors were defined. The 2D error vectors were analysed based on two components: modules (linear statistics) and azimuths (circular statistics). Figure 5 shows the dispersion of the corners. The linear and circular statistics are shown in Table 2.

The confidence interval for the mean module in linear statistics is an estimator of the variability on the location of the corners. As in the previous tests, the results are better in Macro Mode than in Wide Mode. It is observed that the repeatability is slightly greater than the corresponding mode accuracy: ±1.82 *vs.* ±1.66 mm in Wide Mode and ±0.84 *vs.* ±0.81 mm in Macro Mode. Both are sources of error that must be considered together and whose influence depends on the objectives of the scan.

The value of the Von Mises parameter shows that the circular distribution of the error is isotropic in both Macro and Wide Modes (this concentration parameter is significant if its value is greater than 1.5) [22]. No significant differences were detected in any particular direction in terms of repeatability.

## 5. Conclusions

Low-cost 3D laser scanners are a valuable tool for elaborate digital collections of archaeological objects or for analysing the shape and dimensions of fragile pieces. However, it is important to know their potential and their limitations. In this work, the uncertainty and repeatability of a NextEngine scanner was tested. The results show that the uncertainty in the Macro Mode is approximately half that of the Wide Mode and that the accuracy as tested was significantly lower stated in the manufacturer specifications. A systematic error appeared in some cases involving the Z distances, both in Macro and Wide Modes, suggesting the presence of a bias in this direction that must be taken into account if the scan results can be relevant. Other results show a clearly observable difference in position of the object in repetitive scans. The relative dispersion of the observations is slightly larger than the corresponding mode accuracy, and the results show that the circular distribution of the error is isotropic in both Macro and Wide Modes. We emphasise that repeatability is not a key issue in most applications, but the existence of different absolute locations of replicates is a matter that must be known to the user.

We suggest some improvements for the use of this device to generate reliable and accurate digital object collections. The first suggestion is to add an external and calibrated reference scale that allows for measuring the absolute position of scanned data in repetitive operations. For example, two perpendicular planes with a reference frame with point or crosses (similar to patters used to calibrate photographic cameras). The second is to provide a dimensionally calibrated object that allows for a self-calibration operation of the scanner.

The manufacturer recommends preparing dark, shiny or transparent objects using tools such as paint pens, white hairspray or talc. These tools can help the lasers to capture the data but these procedures are forbidden if the object is a valuable archaeological piece. In conclusion, we can assume that the values of uncertainty may worsen when the reflective properties of the object are less favourable.

Finally, some differences in luminosity and colour temperature were observed in the analysis of repeatability. Although we did not analyse the capture of textures, we suggest that a colour scale be provided as well as a protocol for allowing colour correction in post-processing stages.

This scanner is a low-cost, reasonably fast, non-destructive and valuable tool for scanning small objects. Its uncertainty values are more than adequate for a wide set of applications, such as museums, natural history collections, and digital catalogues. We would suggest that these additional values be added to the metadata information about each scanned object.

## Figures and Tables

**Table 1.** The mean and 95% confidence interval of the difference between calliper and scanner measures (mm). *P*-values test the differences between calliper and scanner distances (ns = non-significant). Sample size is *n* = 20. (cited from http://www.graphpad.com/quickcalcs/ttest1.cfm?Format=SD).

**Distance**	**Macro mode**	**P**	**Wide mode**	**P**
BX	−0.03 ± 0.85	ns	−0.01 ± 1.46	ns
BZ	−0.66 ± 0.83	<0.0001	−0.17 ± 1.72	ns
CX	−0.11 ± 0.61	ns	0.00 ± 1.44	ns
CZ	−1.51 ± 0.94	<0.0001	−0.82 ± 2.01	0.004

**Table 2.** Basic linear and circular statistics for the dispersion vectors of the B section corners.

**Parameter**	**Macro Mode**	**Wide Mode**
Sample size	80	80

**Linear statistics (mm)**		

Mean module	0.48	1.28
Module standard deviation	0.43	0.93
Confidence interval (95%)	±0.84	±1.82

**Circular statistics**		

Mean azimuth (°)	54	317
Mean module (vectorial)	0.18	0.12
Von Mises parameter	0.38	0.25
